# Supplementary material for: Bis-naphthopyrone pigments protect filamentous ascomycetes from a wide range of predators
Source: Nat Commun. 2019 Aug 8;10:3579. doi: 10.1038/s41467-019-11377-5 (PMC6687722; doi:10.1038/s41467-019-11377-5)
Supplement: Supplementary file 1 — Supplementary Information [file 41467_2019_11377_MOESM1_ESM.pdf]

SUPPLEMENTARY INFORMATION

Protection of filamentous fungi from wide-range of predators by bis-naphthopyrone pigments

Xu et al.

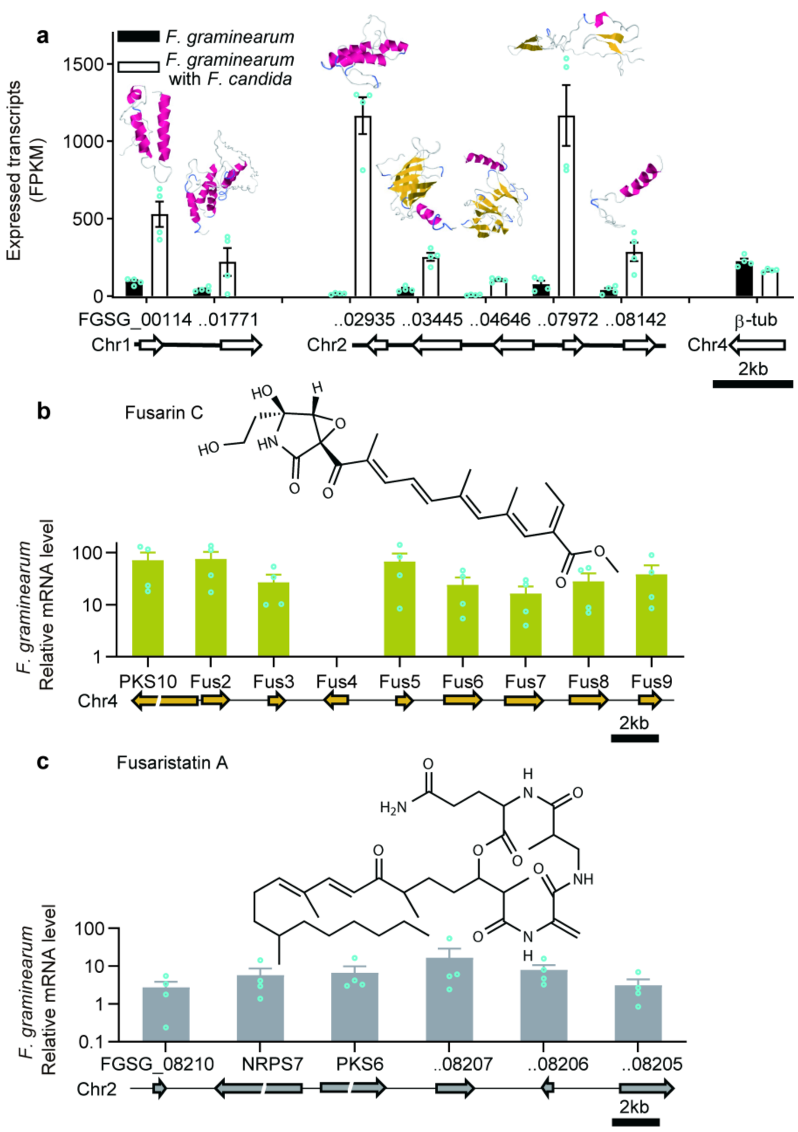

Supplementary Fig. 1 Springtails grazing induces biosynthetic pathways for cysteine-rich proteins, fusarin C and fusaristatin A in *F. graminearum*. **a** Fragments per kilobase of mapped reads (FPKM) generated by Cufflinks for 7 most strongly up-regulated genes encoding small secreted cysteine-rich proteins in *F. graminearum* when predated by the springtail *F. candida* for 48 h according to RNAseq data; beta-tubulin gene is shown as a reference. **b,c** Up-regulation of fusarin C and fusaristatin A pathways in *F. graminearum* after exposure to the springtail *F. candida* for 48 h. The RNAseq analysis was carried out with four biological replicates; error bars show SEM. Source data are provided in a Source Data file.

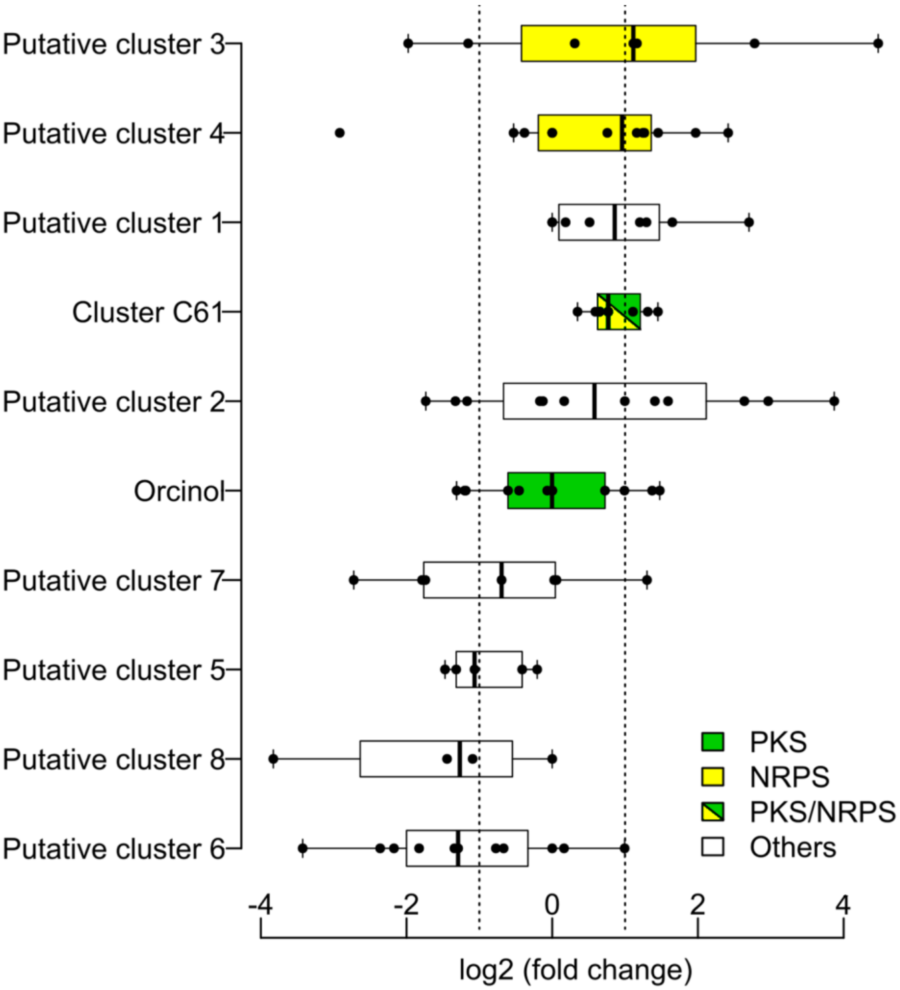

Supplementary Fig. 2 Effect of springtail grazing on the transcription of additional gene clusters encoding secondary metabolite synthesis in *Fusarium graminearum*. *F. graminearum* was exposed to the springtail *F. candida* for 48 h and mRNA levels were determined by RNAseq. Accession numbers: putative cluster 3 (FGSG_03428 to FGSG_03434), putative cluster 4 (FGSG_07820 to FGSG_07831) putative cluster 1 (FGSG_01672, FGSG_01673, FGSG_01675 to FGSG_01677, FGSG_01679 to FGSG_01681), cluster C61 (FGSG_10542, FGSG_10543, FGSG_10545 to FGSG_10547, FGSG_10549, FGSG_13782), putative cluster 2 (FGSG_11984 to FGSG_11989, FGSG_01685 to FGSG_01690), orcinol (FGSG_03956 to FGSG_03959, FGSG_03962 to FGSG_03971), putative cluster 7 (FGSG_09060 to FGSG_09066), putative cluster 5 (FGSG_04173 to FGSG_04177), putative cluster 8 (FGSG_10494 to FGSG_10496,

FGSG_10498), putative cluster 6 (FGSG_04740 to FGSG_04750). The RNAseq analysis was carried out on four biological replicates. Box plots show the median for each cluster and lower and upper quartiles (Q1 and Q3). Up-regulated clusters were defined as having >50% of the genes and/or the gene encoding the signatures enzyme induced [log2 FPKM (fold change) higher than 1.0 (dotted line) and a q value lower than 0.01]. Whiskers show the largest (smallest) observation or 1.5-fold of the interquartile range, whichever is smaller (larger) [Q1-1.5*(Q3-Q1) and Q3+1.5*(Q3-Q1)]. Source data are provided in a Source Data file.

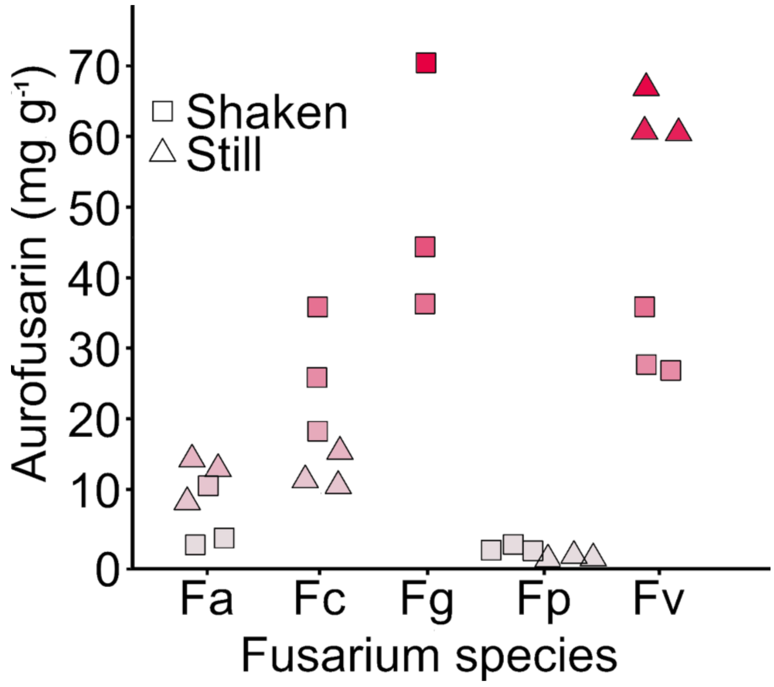

Supplementary Fig. 3 The concentration of aurofusarin in mycelia of *Fusarium avenaceum* BBA92013 (Fa), *F. culmorum* 3.37 (Fc), *F. graminearum* IFA66 (Fg), *F. poae* DSMZ62376 (Fp), and *F. venenatum* RD90 (Fv) were grown in still and shaken (140 rpm) cultures in PDB for 14 d at 23°C, mycelia were harvested by filtration, freeze-dried, extracted with chloroform-methanol (80:20) and aurofusarin content was determined by HPLC-MS/MS. The coloration of symbols indicates aurofusarin concentration in mycelia. Source data are provided in a Source Data file.

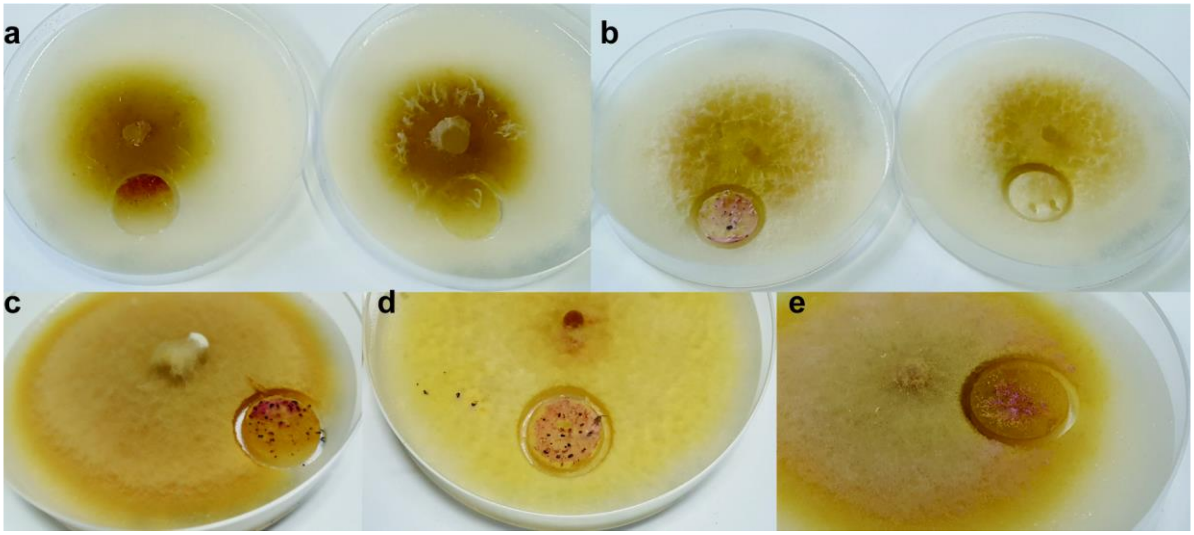

Supplementary Fig. 4 Stimulation of the synthesis of red pigment in *Fusarium* spp. by grazing. **a,** Left: *F. poae* DSMZ 62376 culture on rice agar with an arena exposed to *F. candida* grazing for 2 d; right: control with the same arena without animals. **b,** *F. venenatum* RD15 culture with an arena exposure to *F. candida* grazing for 2 d; right: control with the same arena without animals. **c,** *F. sporotrichoides* IPP 0249 culture with an arena exposed to the isopod *Porcellio scaber* for 2 d. **d,** *F. venenatum* RD 90 culture with an arena exposure to the isopod *Porcellio scaber* for 2 d. **e,** *F. avenaceum* BBA92013 culture with an arena exposure to *F. candida* for 2 d. The animals were confined to grazing arenas of 15 mm diameter with plastic cylinders inserted into the agar; the cylinders and animals were removed before the photos were taken.

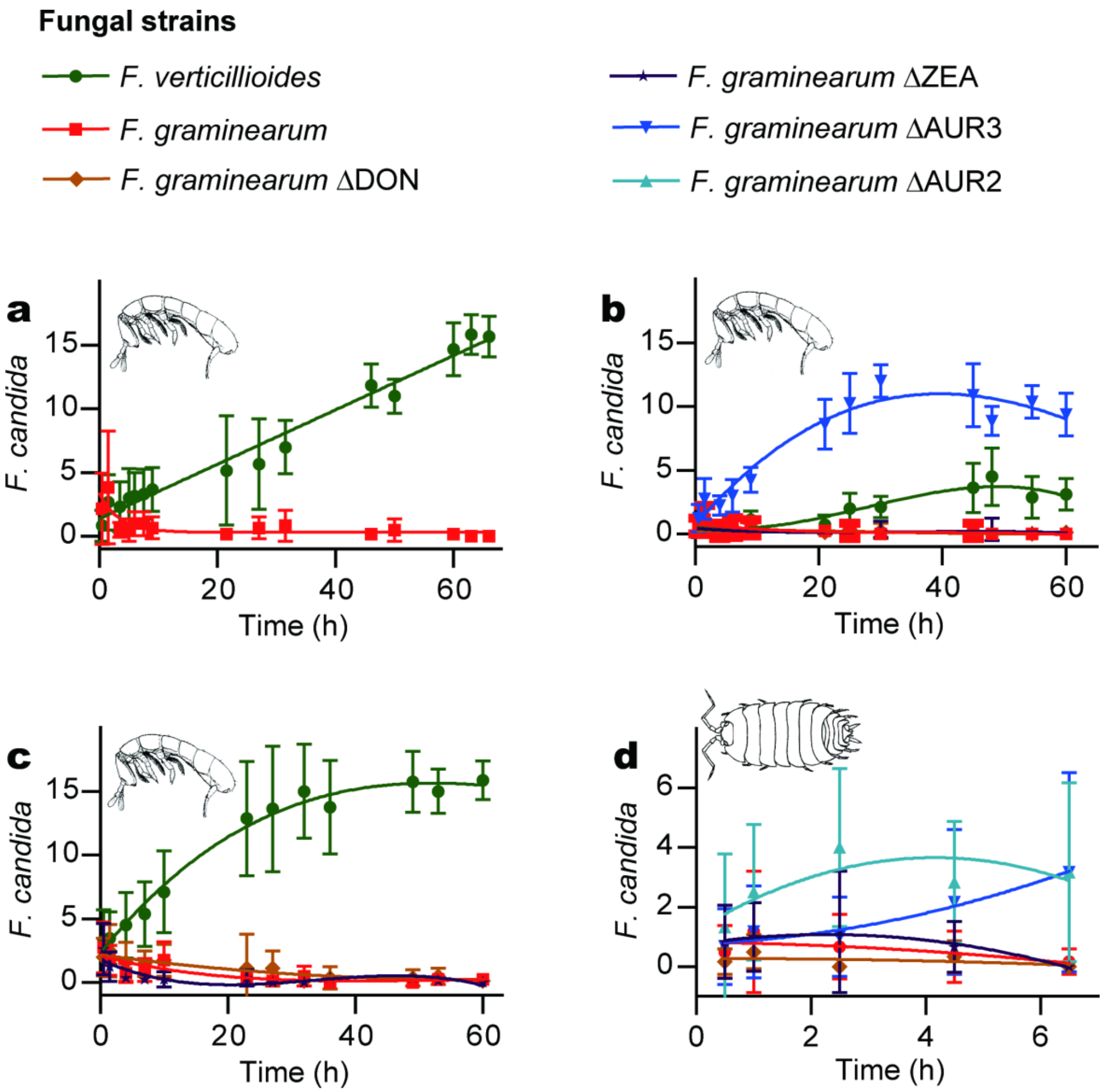

Supplementary Fig. 5 Effect of aurofusarin and mycotoxins deoxynivalenol and zearalenone on the food preference in arthropods. **a** Preference of the springtail *F. candida* for *Fusarium verticil-lioides* over *F. graminearum*. The springtails starved for two days were placed into the centre of a Petri dish with the fungal cultures on the opposite sides of the dish and the number of animals feeding on each culture was monitored (20 animals per plate; 6 replicates). **b** Disruption of aurofusarin synthesis in *F. graminearum* (ΔAUR2, ΔAUR3) reversed the preference of *F. candida* for *Fusarium* species; disruption of zearalenone (ΔZEN) and deoxynivalenol (ΔDON) synthesis did not affect the food preference. The fungal cultures were placed along inner edges of

Petri dishes in equal distances (20 animals per plate; 8 replicates). **c** *F. candida* preferred *F. verticillioides* over *F. graminearum* wild type and *F. graminearum* strains with disrupted synthesis of zearalenone and deoxynivalenol (20 animals per plate; 8 replicates). **d** Isopod *T. tomentosa* preferred *F. graminearum* strains with disrupted synthesis of aurofusarin over wild type strain and strains with disrupted synthesis of zearalenone and deoxynivalenol (8 animals per plate; 6 replicates). Error bars show 95% CI. Source data are provided in a Source Data file.

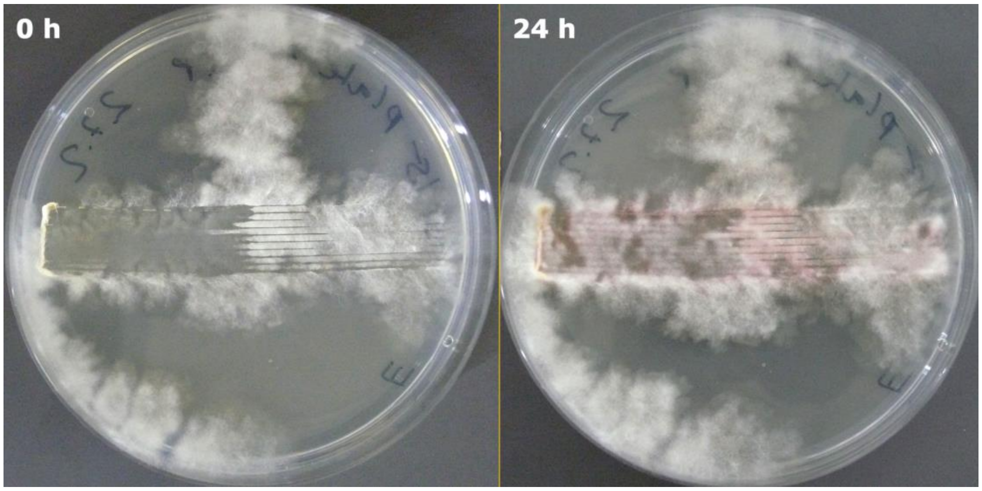

Supplementary Fig. 6 Effect of mechanical damage on *Fusarium venenatum*. Culture of *F. venenatum* RD15 growing on GM7 medium at was injured with a razor blade array as in Fig. 6 and photos were taken immediately after the injury (left) and 24 h later (right).
