# Supplementary material for: Bis-naphthopyrone pigments protect filamentous ascomycetes from a wide range of predators
Source: Nat Commun. 2019 Aug 8;10:3579. doi: 10.1038/s41467-019-11377-5 (PMC6687722; doi:10.1038/s41467-019-11377-5)
Supplement: Supplementary file 3 — Description of Additional Supplementary Files [file 41467_2019_11377_MOESM3_ESM.pdf]

**Description of Additional Supplementary Files**

File Name: Supplementary Data 1
Description: The analysis of the effect of springtail grazing on the expression of 13,710 genes of *Fusarium graminearum* carried out by RNA sequencing (RNAseq).

File Name: Supplementary Data 2
Description: List of PCR primers used.

File Name: Supplementary Data 3
Description: Summary of the statistics with the number of replicates, statistical tests used, and p-values.
