# Supplementary material for: Bis-naphthopyrone pigments protect filamentous ascomycetes from a wide range of predators
Source: Nat Commun. 2019 Aug 8;10:3579. doi: 10.1038/s41467-019-11377-5 (PMC6687722; doi:10.1038/s41467-019-11377-5)
Supplement: Supplementary file 7 — Reporting Summary [file 41467_2019_11377_MOESM7_ESM.pdf]

nature research

Corresponding author(s): Petr Karlovsky

Last updated by author(s): Jun 16, 2019

Reporting Summary

Nature Research wishes to improve the reproducibility of the work that we publish. This form provides structure for consistency and transparency in reporting. For further information on Nature Research policies, see Authors & Referees and the Editorial Policy Checklist.

Statistics

For all statistical analyses, confirm that the following items are present in the figure legend, table legend, main text, or Methods section.

n/a	Confirmed	
☐	☒	The exact sample size (*n*) for each experimental group/condition, given as a discrete number and unit of measurement
☐	☒	A statement on whether measurements were taken from distinct samples or whether the same sample was measured repeatedly
☐	☒	The statistical test(s) used AND whether they are one- or two-sided<br>*Only common tests should be described solely by name; describe more complex techniques in the Methods section.*
☐	☒	A description of all covariates tested
☐	☒	A description of any assumptions or corrections, such as tests of normality and adjustment for multiple comparisons
☐	☒	A full description of the statistical parameters including central tendency (e.g. means) or other basic estimates (e.g. regression coefficient) AND variation (e.g. standard deviation) or associated estimates of uncertainty (e.g. confidence intervals)
☐	☒	For null hypothesis testing, the test statistic (e.g. F, t, r) with confidence intervals, effect sizes, degrees of freedom and P value noted<br>*Give P values as exact values whenever suitable.*
☒	☐	For Bayesian analysis, information on the choice of priors and Markov chain Monte Carlo settings
☒	☐	For hierarchical and complex designs, identification of the appropriate level for tests and full reporting of outcomes
☒	☐	Estimates of effect sizes (e.g. Cohen's *d*, Pearson's *r*), indicating how they were calculated

Our web collection on statistics for biologists contains articles on many of the points above.

Software and code

Policy information about availability of computer code

Data collection	No software was used for data collection
Data analysis	RNAsec experiments were evaluated using Galaxy version 2018. The data were mapped to F. graminearum genome using Hisat2 v2.0.5.1 and evaluated using Cufflinks v2.2.1 and Cuffdiff v2.2.1.3. AntiSMASH 3.0 was used for the analysis of secondary metabolite pathways. The sources and references for the software are listed in Material and Methods. RT qPCR data were processed using CFX Maestro 1.1 (BioRad). GraphPad Prism 8 was used for statistical tests and to generate charts and graphs.

For manuscripts utilizing custom algorithms or software that are central to the research but not yet described in published literature, software must be made available to editors/reviewers. We strongly encourage code deposition in a community repository (e.g. GitHub). See the Nature Research guidelines for submitting code & software for further information.

Data

Policy information about availability of data

All manuscripts must include a data availability statement. This statement should provide the following information, where applicable:

- Accession codes, unique identifiers, or web links for publicly available datasets
- A list of figures that have associated raw data
- A description of any restrictions on data availability

All data are available in the manuscript and the associated Supplementary Data and Data Source files. The RNAseq data have been uploaded to a public repository as specified in th data availability statement in the manuscript. There is no restriction on data availability.

Field-specific reporting

Please select the one below that is the best fit for your research. If you are not sure, read the appropriate sections before making your selection.

☒ Life sciences ☐ Behavioural & social sciences ☐ Ecological, evolutionary & environmental sciences

For a reference copy of the document with all sections, see nature.com/documents/nr-reporting-summary-flat.pdf

Life sciences study design

All studies must disclose on these points even when the disclosure is negative.

Sample size	In the RNAseq experiment, four biological replicates were used because the large sampling depth of RNAseq allows for the identification of strongly induced genes already with 3 replicates. The key results were generated in food choice experiments; the sample size was chosen according to practical considerations (space for the arenas and time needed to take a photo of each sample at each time interval). The chosen sample size turned out fully sufficient for the purpose because the magnitude of the observed effects was so large that statistical tests were deemed unnecessary.
Data exclusions	No data have been excluded based on measured values; occasionally spontaneously contaminated arenas or agar media were discarded.
Replication	Apart from the replicates described in the manuscrpit and in associated data files, the food choice experiments have been repeated by another person (a laboratory technician) independently, leading to essentially the same results. Furthermore, observation of the same repellent effect of aurofusarin in numerous animal species, of similar induction of red pigment by predation in several Fusarium species, and of the same repellent effect exerted by three bis-naphthopyrones (aurofusarin, viomellein and xanthomegnin) are replications with different animals, fungi and metabolites, which support the general validity of our findings.
Randomization	In food choice and fitness experiments with animals, the arenas were randomize to prevent the effect of light intensisty (for experiments that have not been carried out in darkness), temperature or other environmental conditions.
Blinding	Blinding was not deemed necessary. The only measurements that were not generated by machines was the location of animals on food sources in food choice experiment. To prevent any individual/subjective bias, the experiments were evaluated using photographs that had been taken for each arena at each time point. (These photographs are available for re-check.)

Reporting for specific materials, systems and methods

We require information from authors about some types of materials, experimental systems and methods used in many studies. Here, indicate whether each material, system or method listed is relevant to your study. If you are not sure if a list item applies to your research, read the appropriate section before selecting a response.

Materials & experimental systems

n/a	Involved in the study
☒	☐ Antibodies
☐	☒ Eukaryotic cell lines
☒	☐ Palaeontology
☐	☒ Animals and other organisms
☒	☐ Human research participants
☒	☐ Clinical data

Methods

n/a	Involved in the study
☒	☐ ChIP-seq
☒	☐ Flow cytometry
☒	☐ MRI-based neuroimaging

Eukaryotic cell lines

Policy information about cell lines

Cell line source(s)	Spodoptera frugiperda 9. The cell line was obtained from ATCC.
Authentication	ATCC uses morphology, karyotyping and PCR based approaches to identify cell lines.
Mycoplasma contamination	Mycoplasma testing of cell line is tested regularly to ensure that the results are obtained from mycoplasma non-contaminated cells.
Commonly misidentified lines (See ICLAC register)	Sf9 was the only cell line used. This cell line is not commonly misidentified.

Animals and other organisms

Policy information about studies involving animals; ARRIVE guidelines recommended for reporting animal research

Laboratory animals	Species: Tenebrio molitor, Trichorhina tomentosa, Folsomia candida (strain: Berlin), Drosophila melanogaster, Aphelenchoides saprophilus, Bursaphelenchus mucronatus. The sex of the animals was not determined and except for F. candida, animal cultures have not been initiated from or assigned to designated strains.
Wild animals	Isopod (woodlice) Porcellio scaber was collected from below stones and wood residues by soft forceps and transported to the lab in a plastic box filled with soil and leaves. The sex and age of the animals were not determined. After the study, the animals were kept in culture for future experiments.
Field-collected samples	Woodlice were kept in a plastic box filled with moist soil covered with leaves. The box was kept in a dark place at room temperature. A few pieces of raw potato or carrot were added as food at irregular intervals, and the soil was kept moist by adding tap water as needed.
Ethics oversight	No ethical approval was required for work with nematodes, isopods and insects.

Note that full information on the approval of the study protocol must also be provided in the manuscript.
